# Reliability and validity of clinically accessible smartphone applications to measure joint range of motion: A systematic review

**DOI:** 10.1371/journal.pone.0215806

**Published:** 2019-05-08

**Authors:** Justin W. L. Keogh, Alistair Cox, Sarah Anderson, Bernard Liew, Alicia Olsen, Ben Schram, James Furness

**Affiliations:** 1 Faculty of Health Sciences and Medicine, Bond University, Gold Coast, Queensland, Australia; 2 Sports Performance Research Institute New Zealand (SPRINZ), AUT University, Auckland, New Zealand; 3 Cluster for Health improvement, Faculty of Science, Health, Education and Engineering, University of the Sunshine Coast, Sunshine Coast, Queensland, Australia; 4 Department of Physiotherapy, Faculty of Health Sciences and Medicine, Bond University, Gold Coast, QLD, Australia; 5 Centre of Precision Rehabilitation for Spinal Pain (CPR Spine), School of Sport, Exercise and Rehabilitation Sciences, University of Birmingham, Edgbaston, Birmingham, United Kingdom; 6 Water Based Research Unit, Bond Institute of Health and Sport, Bond University, Gold Coast, QLD, Australia; Hochschule Trier, GERMANY

## Abstract

Measuring joint range of motion is an important skill for many allied health professionals. While the Universal Goniometer is the most commonly utilised clinical tool for measuring joint range of motion, the evolution of smartphone technology and applications (apps) provides the clinician with more measurement options. However, the reliability and validity of these smartphones and apps is still somewhat uncertain. The aim of this study was to systematically review the literature regarding the intra- and inter-rater reliability and validity of smartphones and apps to measure joint range of motion. Eligible studies were published in English peer-reviewed journals with full text available, involving the assessment of reliability and/or validity of a non-videographic smartphone app to measure joint range of motion in participants >18 years old. An electronic search using PubMed, Medline via Ovid, EMBASE, CINAHL, and SPORTSDiscus was performed. The risk of bias was assessed using a standardised appraisal tool. Twenty-three of the eligible 25 studies exceeded the minimum 60% score to be classified as a low risk of bias, although 3 of the 13 criteria were not achieved in >50% of the studies. Most of the studies demonstrated adequate intra-rater or inter-rater reliability and/or validity for >50% of the range of motion tests across all joints assessed. However, this level of evidence appeared weaker for absolute (e.g. mean difference ± limit of agreement, minimal detectable change) than relative (e.g. intraclass correlation, correlation) measures; and for spinal rotation than spinal extension, flexion and lateral flexion. Our results provide clinicians with sufficient evidence to support the use of smartphones and apps in place of goniometers to measure joint motion. Future research should address some methodological limitations of the literature, especially including the inclusion of absolute and not just relative reliability and validity statistics.

## Introduction

The measurement of joint range of motion (ROM) in static and dynamic, passive and active, human movements is an essential skill in the musculoskeletal assessments commonly performed by physiotherapists, as well as some strength and conditioning coaches, to examine joint function, detect joint asymmetry and evaluate treatment efficacy as an objective outcome measure [1]. In the present study, static ROM is defined as the range of a joint held motionless at either of its limit of movement. Dynamic ROM is the range a joint moved to and from the limits of movement. When a joint is moved passively by an assessor or external device, passive ROM is assessed. When a joint moves as a result of muscular contraction, active ROM is assessed. The universal goniometer has long been the preferred method of clinical ROM measurement (especially static ROM) due to its ease of use, low cost, and demonstrated reasonable levels of reliability and validity in numerous studies [2–4].

However, the universal goniometer is not without its drawbacks, even when assessing static joint ROM. When assessing static ROM such as the angle of hinge joints like the knee and elbow in adults, there may always be some degree of error due to the universal goniometer not typically being long enough to be aligned directly with the appropriate landmarks on both proximal and distal adjacent joints. Spinal rotation may also be difficult to measure with a universal goniometer due to the difficulty in palpating anatomical landmarks to use as a reference point [5–7]. It is perhaps no surprise then that reliability is reduced when measuring the spinal compared to upper and lower limb motion with a universal [6–9]. These potential issues highlighted for the use of the universal goniometer in assessing static joint ROM may be further exacerbated in inexperienced clinicians who have a relative inability to correctly locate anatomical landmarks; as well as the assessment of dynamic rather than static ROM [10, 11].

The development of smartphone technology and software applications (apps), coupled with the ubiquity of smartphone ownership, now allows smartphones to measure joint ROM. Like the universal goniometer, smartphones are similarly easy to use, relatively inexpensive, and highly accessible [12]. Their inbuilt sensors such as an accelerometer, gyroscope, and magnetometer provide the necessary equipment to allow the smartphone to measure angles and displacements [12]. With the use of apps that can be downloaded onto the smartphone, these measurements can be transformed into meaningful assessment data such as joint ROM. One possible advantage of smartphone apps is that their use may circumvent some of the difficulties of using the universal goniometer regarding landmark identification and alignment. Where smartphone apps can altogether overcome the aforementioned drawbacks of the universal goniometer may depend upon the technology used and the experience of the clinician with this alternative approach. The emergence of smartphone apps therefore presents the clinical practitioners with a new set of tools to incorporate into clinical practice, especially for some of the more difficult joint ROMs to quantify.

In order for clinicians to be willing to replace the universal goniometer (at least in some contexts) with smartphone apps as a tool to clinically assess ROM, the validity and reliability of smartphone apps must be comparable or better than the universal goniometer. In psychometric terminology, reliability deals with the consistency in angle and displacement measures produced by smartphone apps, when used by multiple assessors (inter-rater), and when the same assessor performs multiple measurements (intra-rater) [13]. Validity deals with the extent that the measurement obtained from one device, such as smartphone apps, correlates or matches the criterion laboratory devices such as 3-D motion capture or criterion clinical tools such as the universal goniometer [13].

On the topic of synthesizing the psychometric properties of smartphone apps, a number of systematic reviews have been conducted [14, 15]. However, the review of Milani et al. [14] is considered to be outdated due to the relative explosion of research into human movement analysis apps and as such, only included 12 studies assessing joint angle measurements. Further, while the review of Rehan Youssef and Gumaa [15] was more recent and well conducted in most aspects, there were several methodological limitations. First, their literature search was completed in August 2016 (including 15 studies and one case study assessing joint ROM) [15]. Second, they utilised a non-validated risk of bias assessment tool that they personally developed [15]. Third, there was a relative lack of reporting of specific reliability and validity data for each of the multiple actions that can occur at some joints such as the spine (trunk) and shoulder joints [15]. The relative lack of reporting specific data for each joint action is a major issue for clinicians, as it is quite possible that a particular smartphone and app may have sufficient reliability and/or validity or measuring some actions at a particular joint in certain planes of motion (e.g. flexion and extension in the sagittal plane) but that more complicated actions such as rotation in the transverse plane may be less reliable and/or valid.

The purpose of this systematic review was to address some of the limitations of the previous review in this area so as to better assist the clinician identify which smartphone apps may show adequate inter-rater and intra-rater reliability as well as validity for the measurement of ROM at particular joints and actions in clinical practice. This state-of-the-art review will assist clinical practitioners in deciding the appropriateness and choice of smartphone apps for clinical ROM assessment.

## Search methodology

### Search strategy

The protocol for this systematic review has not been registered. A database search of PubMed, Medline via Ovid, EMBASE, CINAHL, and SPORTSDiscus was initially performed on 20th October 2017 by two independent reviewers. This search was repeated on 20th December 2018 to maximise the currency of the findings of this review. The search strategy is described in Appendix 1.

#### Inclusion and exclusion criteria

Studies retrieved from the search process were determined by two independent reviewers, with a third reviewer used to assist with consensus, were any discrepancies being initially reported by the first two independent reviewers. The eligibility of the studies to be included in this review was determined by the following criteria: published in peer-reviewed journals; measure human participants aged over 18 years old; used a smartphone app to measure joint ROM and assessed validity and/or reliability of these apps; published from 2007 as this was the year the iPhone was launched; published in English and have full text available. Case studies, abstracts only or grey literature were not included. Smartphone apps which required either image/video recordings and/or post data collection analyses to generate joint angles were excluded, as such an approach is unlikely to be used in clinical practice due to privacy concerns with the storage of video footage and the additional analysis time that would be required.

Quality assessment

The Critical Appraisal Tool (CAT), developed by Brink and Louw [16] was used to appraise the methodological quality of studies reporting a reliability and/or validity component. The included studies to be appraised were rated on a set of specific criteria involving 13 items that assessed a variety of methodological factors including subject and rater characteristics, rater blinding, testing order, criterion measure characteristics and statistical analyses performed [16]. Consistent with a recent study that has used the CAT [17], in order to satisfy Criteria 13 (Statistical Methods) the study had to report absolute reliability and/or validity statistics (e.g. SEM, MDC or MD±LOA) in addition to the more commonly reported relative reliability and/or validity statistics (e.g. r or ICC). As not all included studies assessed validity, not all the CAT criteria were relevant to each study. In this case, each validity item was scored as not applicable (NA) and that criteria not included in the overall assessment of the particular study’s risk of bias. Consistent with the use of the CAT in previous studies, a threshold of ≥ 60% was considered as high quality, and a quality of < 60% was rated as poor quality, consistent with previous systematic reviews [4, 17, 18].

The methodological quality of the studies identified by the search was assessed by two independent reviewers. Across all the 13 items of the CAT, there was an overall agreement of 86.2% between the raters when reviewing the methodological quality of the 37 articles included in this review, resulting in a Cohen’s unweighted Kappa statistic of 0.64, indicating good agreement between the two raters [19].

Data extraction

Data was obtained from studies that met the inclusion and exclusion criteria, which included: the CAT assessment, participants, application and smartphone device, joint movement assessed and position that the participant was in whilst being assessed. Where applicable, data was extracted for intra-rater and inter-rater reliability as well as validity. Both relative and absolute reliability and validity statistics were reported where available to provide an index of the correlation or rank order (relative measure) and change/difference in the mean (absolute measure) [20, 21]. Common measures of relative reliability and validity include the intra-class correlation coefficient (ICC), concordance correlation coefficient (CCC) and Pearson’s product moment correlation (r). Alternatively, common measures of absolute reliability and validity include the standard error of measurement (SEM), minimal detectable change (MDC), mean difference (MD) and limits of agreement (LoA).

Data analysis

A critical narrative approach was applied to synthesize and analyse the data. For each measure, the following criteria were used to judge the level of intra-rater and inter-rater reliability and validity. For relative measures, the following criteria were used ICC: Poor = ICC < 0.40, Fair = ICC 0.40–0.59, Good = ICC 0.60–0.74, Excellent ≥ 0.75 [22]; for r: negligible r = 0–0.29, low r = 0.30–0.49, moderate r = 0.50–0.69, high r = 0.70–0.89, very high r = 0.90–1 [23]; and for CCC: Poor CCC < 0.90, Moderate CCC = 0.90–0.94, Substantial CCC = 0.95–0.99, Almost perfect CCC > 0.99 [24]. For absolute measures of reliability and validity, the following criteria were used SEM: Poor SEM > 5° and Good SEM ≤ 5° [25]; for MDC: Poor MDC > 5° and Good MDC ≤ 5° [25, 26] for LOA, a standard deviation threshold of 5° [25–28] multiplied by 1.96 to derive the 95% LOA bandwidth: poor > ± 9.8° and Good < ± 9.8°.

## Results

### Selection of studies

**Fig 1:**
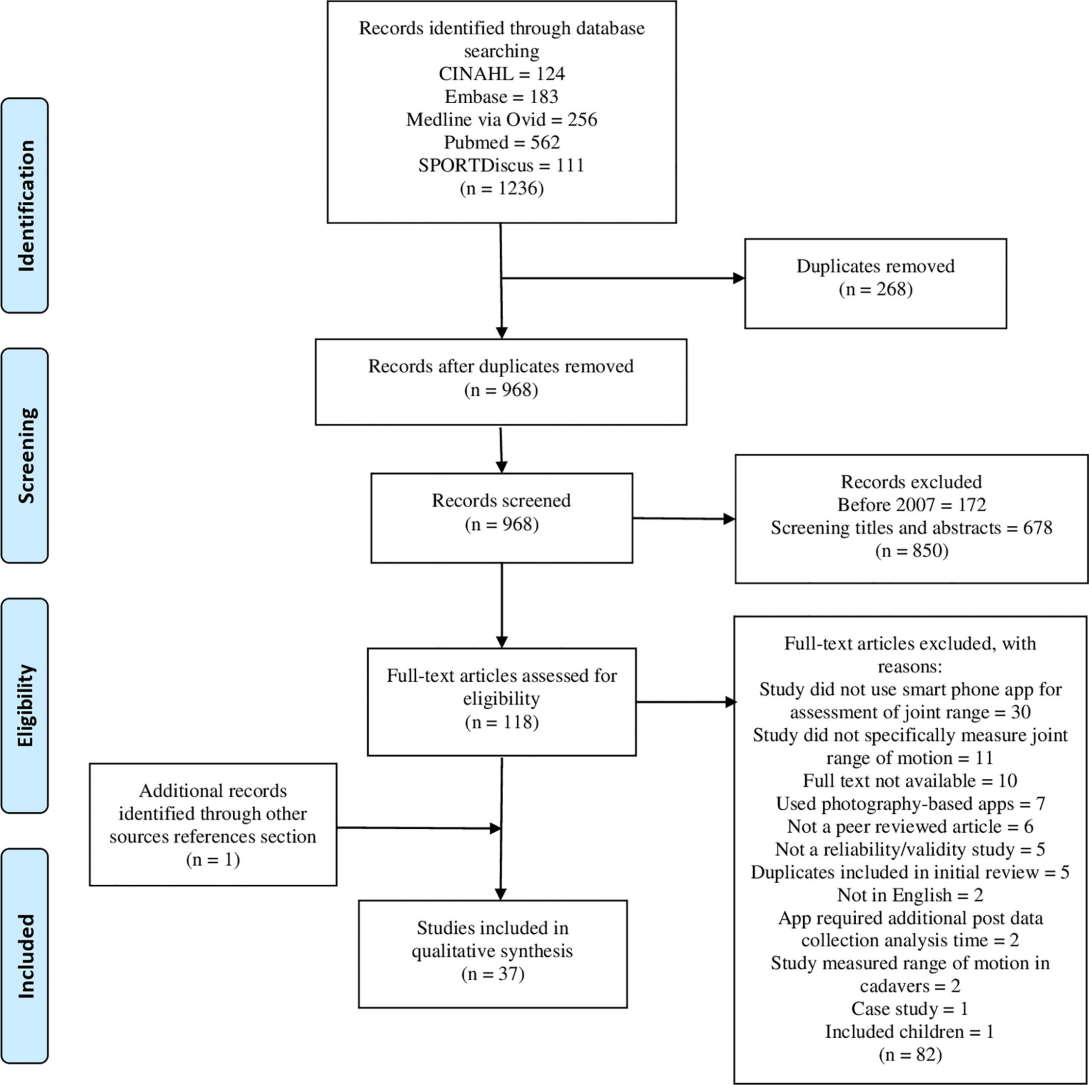
PRISMA flow chart of the screening process.

Fig 1 represents the article review process based on the Preferred Reporting Items for Systematic Reviews and Meta-Analyses (PRISMA) guidelines [29]. Our initial literature search identified 1066 studies, with the second literature search identified an additional 170 studies, leading to combined total of 1236 identified studies. Within the 1236 identified studies, 268 duplicates were removed prior to the title and abstract screening, with an additional five duplicates subsequently identified when screening the second literature search. The search strategy yielded 36 eligible studies, with one additional study identified through other sources for a total of 37 studies.

### Study characteristics and methodology

A description of the broad methodology of each included study was depicted in Table 1. In order of the most common to least common joints assessed, were spine (trunk), knee, shoulder, wrist, elbow, ankle and hip. As the trunk and shoulder allow movement in more directions than other joints, the studies assessing the trunk and shoulder typically looked at a greater number of joint movements across the multiple planes of movement. For example, the studies assessing trunk motion typically looked at trunk flexion/extension, lateral flexion and axial rotation; with the studies assessing shoulder motion typically examining flexion, abduction, horizontal adduction as well as external/internal rotation.

**Table 1:** Characteristics of studies included in this review.

Reference	Participants	Movement assessed	Position	Application	Device	Criterion instrument
**Spine (trunk)**						
Bedekar et al. [38]	30 healthy students (5 males and 25 females, age 21.5 ± 1.5 years).	Active, static lumbar flexion	Standing	Goniometer	iPod (model not stated)	Dual inclinometer
Furness et al. [39]	30 healthy students (20 females, 10 males; age: 29.8 ± 8.9 years)	Active, static thoracic rotation	Seated	Compass app	IPhone 6S	Universal goniometer
Grondin et al. [40]	22 healthy adults (15 male, 7 female, age: 29.9 ± 5.4 years)	Active, static cervical flexion-extension ROM Passive, static cervical flexion-rotation test	Sitting and supine	Clinometer	IPhone 5	
Jung et al. [41]	17 male adults (age: 22.2 ± 1.6 years)	Active, static, pelvic rotation	Supine	Clinometer level and slope finder (Plaincode Software Solutions, Stephanskirchen, Germany)	Not reported	Vicon 3D Motion Analysis
Kolber & Hanney [42]	30 healthy adults (12 male, 18 female, age: 25.6 ± 2.1 years)	Active, static thoracolumbo-pelvic flexion, isolated lumbar flexion, thoracolumbo-pelvic extension, right lateral flexion, left lateral flexion	Standing	iHandy	IPhone 4	Bubble inclinometer
Pourahmadi et al. [43]	30 healthy adults (15 male, 15 female, age: 27.9 ± 6.3 years)	Active, static lumbar flexion & extension	Standing	The TiltMeter	IPhone 5	Gravity based inclinometer
Pourahmadi et al. [30]	40 adults with non-specific neck pain (20 male, 20 female, age: 31.1 ± 6.4 years)	Active, static cervical flexion, extension, lateral flexion, rotation	Sitting	G-pro	IPhone 7	Universal goniometer
Quek et al. [44]	21 healthy adults (11 male, 10 female, age 31.0 ± 9.1 years)	Active, static cervical ROM (flexion, extension, right lateral flexion, left lateral flexion, right rotation, left rotation.	Sitting	Custom-built app designed by a co-author (RC) of this study using MIT App Inventor.	Samsung Galaxy S3	Vicon 3D Motion Analysis
Stenneberg et al. [31]	Validity study 30 patients with neck pain (19 female, 11 male, age: 53.4 ± 9.1 years) Reliability study 26 patients with neck pain (19 female, 7 male, age: 45.2 ± 15.3 years)	Active, static cervical ROM (flexion- extension, lateral flexion, rotation)	Sitting	3D range of motion	IPhone 4s	Polhemus 3D Motion Analysis
Tousignant-Laflamme et al. [45]	28 healthy adults (9 male, 19 female, age 23 ± 6 years)	Active, static cervical ROM (flexion, extension, right lateral flexion, left lateral flexion, right rotation, left rotation.	Sitting	Clinometer (frontal and sagittal planes) Compass (transverse plane)	IPhone (model not stated)	Cervical range of Motion Device (CROM)
Ullucci et al. [46]	38 healthy adults (19 female, 19 male, age: 28 ± 1.2 years)	Passive cervical flexion and right and left rotation	Sitting	Clinometer (plaincode, Stephanskirchen, Germany)	IPhone (model not stated) & Android (model not stated)	
**Shoulder**						
Lim et al. [47]	47 healthy adults (28 male, 19 female, age 24.9 ± 3.5 years)	Passive, static shoulder horizontal adduction	Supine vs. sidelying position	Goniometer Pro	IPhone 5	
Mejia-Hernandez et al. [33]	75 patients (21 female, 54 male, age: 46 years (range, 24–94 years) with shoulder disorders	Forward flexion, Total active abduction, Active glenohumeral Abduction, Passive glenohumeral Abduction, Active internal rotation, Passive internal rotation, Active external rotation, Passive external rotation	Seated and supine	GetMyROM	IPhone 5s	Universal goniometer
Mitchell et al. [48]	94 healthy adults (37 male, 57 female, age: 26.4 ± 7.6 years)	Active, static shoulder external rotation	Supine	GetMyROM	IPhone 4	Standard goniometer
Ramkumar et al. [49]	10 healthy adults (5 male, 5 female, age 27 years)	Active, static, flexion, abduction, internal and external rotation	Not reported	Built-in iPhone sensors	IPhone (model not reported	Standard goniometer
Shin et al. [27]	41 patients with unilateral symptomatic shoulders (20 males, 21 females, age: 52.7 ± 17.5 years).	Active and passive, static shoulder ROM: forward flexion, abduction, external rotation, external rotation at 90^0^ abduction, and internal rotation.	Standing	Clinometer Application, Clinometer-Level and Slope Finder (Plaincode Software Solutions)	Samsung Galaxy S	Standard goniometer
Werner et al. [28]	24 healthy adults (9 male, 15 female) 15 symptomatic adults (all undergone total shoulder replacement 6–12 weeks earlier)	Static abduction, forward flexion, external rotation with arm at side, external rotation with shoulder abducted to 90^0^, internal rotation with arm abducted at 90^0^. Passive/ active ROM not reported	Standing and supine	Smartphone clinometer (Plaincode Software Solutions)	IPhone (model not stated)	Standard goniometer
**Elbow**						
Behnoush et al. [50]	60 healthy adults (47 male, 13 female, age: 42.3 ± 11.4 years).	Active, static elbow flexion, supination, and pronation.	Sitting	Bubble inclinometer	HTC (model not stated)	Universal goniometer
Cruz & Morais [51]	41 healthy adults (21 male, 20 female, age: 31.3 ± 13.2 years)	Passive, static ULNT1 sequence	Supine	Compass	IPhone 4	
Vauclair et al. [52]	30 healthy adults (11 females, 9 males, age: 52 years (range 21–74))	Active, static flexion extension, pronation, supination	Sitting	Clinometer	Not reported	Standard goniometer
**Wrist**						
Lendner et al. [53]	306 wrists from 171 healthy participants (50% male, 50% female wrists, age: 45.9 ± 20.2 years)	Static, wrist flexion-extension & ulnar-radial deviation. Passive/ active ROM not reported	Sitting	Gyroscope	IPhone 4	Goniometer
Modest et al.[35]	30 wrist-injured subjects (age: 47 ± 19 years) 30 wrist-healthy subjects (age: 38 ± 15 years)	Active, static wrist flexion, extension, pronation, supination	Sitting and standing	In built goniometer	IPhone 5	Universal goniometer
Pourahmadi et al. [54]	120 wrists from 70 healthy adults (38 male, 32 female, age: 27.5 years)	Active, static wrist flexion, extension radial deviation and ulnar deviation	Sitting with the forearm placed on a supporting surface	G-pro	IPhone 5	Universal goniometer
Santos et al. [34]	20 healthy adults (10 male, 10 female, age: 52.5 ± 15.5 years) 20 participants with upper limb injuries (10 male, 10 female, age: 41.5 ± 15.7 years)	Active, static forearm pronation and supination	Sitting	Gyroscope, with and without selfie-stick	IPhone 4	Goniometer
**Hip**						
Charlton et al. [55]	20 healthy male adults (age: 23.8 ± 4.6 years)	Passive, static flexion, abduction, adduction, supine internal and external rotation.	Supine: hip flexion, internal and external rotation. Sidelying: abduction, adduction. Seated: seated hip internal and external rotation.	Hip ROM Tester, designed by a co-author (RC) of this study using MIT App Inventor.	Samsung Galaxy S2	Vicon 3D motion capture
**Knee**						
Derhon et al. [56]	34 healthy females (age: 21 ± 2 years)	Passive, static knee extension	Supine	free ROM	Samsung Galaxy S5 smartphone	
Dos Santos et al. [57]	34 healthy females (age: 21 ± 2 years)	Passive, static knee extension	Supine	free ROM	Samsung Galaxy S5 smartphone	Universal goniometer
Hambly et al. [58]	96 healthy adults (79 male, 17 female, age: 31 ±11 years)	Active, static maximum knee joint angle	Supine	iGoniometer	IPhone 3GS	
Hancock et al. [59]	3 healthy adults (absent demographics)	Passive, static knee flexion	Supine	Goniometer Pro	IPhone 7 Plus	
Jones et al. [60]	36 healthy adults (8 male, 28 female, age: 60.6 ± 6.2 years)	Active, static knee joint angle during a lunge	Standing lunge	Simple Goniometer	IPhone 3GS	Universal goniometer
Mehta et al. [36]	60 Orthopedic clinic patients (22 male, 38 female, age: 62.9 ± 8.9 years)	Active, static knee flexion-extension	Supine	i-Goni	IPhone (model not stated)	Universal goniometer
Milanese et al. [1]	6 healthy adults (3 male, 3 female)	Passive, static knee flexion	Supine	Knee Goniometer	IPhone 4	Universal goniometer
Ockendon & Gilbert [61]	5 healthy males, age: 30–40 years	Passive, static knee flexion	Supine with simulated fixed-flexion deformity.	Knee Goniometer	IPhone 3GS	Conventional goniometer
Pereira et al. [37]	20 healthy adults 2 groups of 20 adults post-operative knee surgery Group 1: 8 male, 12 female, age: 72.3 ± 8.8 years, hospitalized 8.5 ± 7.4 days; Group 2: 6 male, 14 female, 72.9 ± 8.9 years, hospitalized 6.9 ± 5.1 days)	Active & passive, static knee flexion & extension	Supine	Knee goniometer	IPhone 4S	Standard goniometer
**Ankle**						
Morales et al. [62]	33 healthy older adults (age: 71 ± 3.6 years)	Active, static ankle dorsiflexion	Weight-bearing lunge test	Inclinometer	IPhone 5S	
Vohralik et al. [63]	20 healthy adults (7 male, 13 female, age: 22.4 ± 2.0 years).	Active, static ankle dorsiflexion	Weight-bearing lunge test	iHandy Level	IPhone (model not stated)	Digital inclinometer and Fastrak 3D motion capture
Williams et al. [64]	20 healthy adults (4 male, 16 female, age: 40 ± 12 years)	Active, static ankle dorsiflexion	Weight-bearing lunge test	Tiltmeter app	IPhone 4 and iPhone 4S	Digital inclinometer

MTPJ = metatarsophalangeal joint, ROM = range of motion, ULNT1 = Upper Limb Neurodynamic Test 1.

The majority of studies involved healthy participants, although some studies involved patients with neck pain [30, 31], shoulder pathology [27, 32, 33], various upper limb injuries [34, 35] or knee pain [36, 37]. A relatively wide variety of smartphones, applications and criterion devices (for the assessment of validity) were utilised in the studies. The most common smartphones were iPhones which were used in 28 studies, with the most common model being the iPhone 4 which was used in nine studies. Samsung phones were used in another six studies, with one study also using an iPod. A wide variety of apps were utilised, with only the most frequently used being the Clinometer (n = 5) and Knee Goniometer (n = 3). All other apps were used in either one or two studies. For the 30 studies that looked at some aspects of validity, the validity of the app was most commonly compared to goniometers (n = 19), 3D motion capture (n = 5) or inclinometers (n = 4).

### Critical appraisal

A critical appraisal of the included articles is summarised in Table 2. The percentage of CAT score ranged from 55% [65] to 100% [32]. Papers with ‘NA’ in their appraisal were not assessed against that particular criteria. Two studies were considered to be of low quality with a score < 60% [53, 55], with another one study close to this criteria with an overall quality score of 62% [38]. Only two of the CAT criteria were achieved in less than 50% of the studies (Criteria Six: Order of Examination and Criteria 13: Statistical Methods). This contrasted with one other criteria being achieved in all studies Criteria #10: Execution of the Index Test).

**Table 2:** Critical appraisal of the eligible studies.

Critical Appraisal Tool (CAT) for Validity and Reliability														
	1 V+R	2 V+R	3 V	4 R	5 R	6 R	7 V	8 R	9 V	10 V+R	11 V	12 V+R	13 V+R	%
**Spine (trunk)**														
Bedekar et al. [38]	Y	Y	N	Y	N	N	Y	Y	Y	Y	N	Y	N	62%
Furness et al. [39]	Y	Y	Y	Y	N	Y	Y	Y	Y	Y	Y	Y	Y	92%
Grondin et al. [40]	Y	Y	NA	NA	Y	Y	NA	N	NA	Y	NA	Y	Y	88%
Jung et al. [41]	Y	Y	Y	NA	N	N	Y	Y	Y	Y	Y	Y	Y	75%
Kolber & Hanney [42]	Y	Y	Y	Y	Y	N	Y	Y	Y	Y	Y	Y	N	85%
Pourahmadi et al. [43]	Y	Y	Y	Y	Y	N	Y	Y	Y	Y	Y	Y	Y	92%
Pourahmadi et al. [30]	Y	Y	Y	Y	Y	N	Y	Y	Y	Y	Y	Y	N	85%
Quek et al. [44]	Y	Y	Y	NA	Y	Y	Y	Y	N	Y	Y	N	Y	83%
Stenneberg et al. [31]	Y	Y	Y	Y	NA	Y	N	N	Y	Y	Y	N	Y	75%
Tousignant-Laflamme et al. [45]	Y	Y	Y	N	N	N	Y	Y	Y	Y	Y	Y	N	69%
Ullucci et al. [46]	Y	Y	NA	Y	Y	Y	NA	Y	NA	Y	NA	Y	N	89%
**Shoulder**														
Lim et al. [47]	Y	Y	NA	Y	Y	Y	NA	Y	NA	Y	NA	Y	N	89%
Mejia-Hernandez et al. [33]	Y	Y	N	Y	NA	N	Y	Y	Y	Y	N	Y	N	67%
Mitchell et al. [48]	Y	Y	Y	Y	Y	Y	Y	Y	Y	Y	Y	Y	N	92%
Ramkumar et al. [49]	Y	N	Y	NA	NA	N	Y	Y	Y	Y	Y	Y	N	73%
Shin et al. [27]	Y	Y	NA	Y	Y	N	NA	Y	NA	Y	NA	Y	Y	89%
Werner et al. [28]	Y	Y	Y	Y	NA	Y	Y	Y	Y	Y	Y	Y	Y	100%
**Elbow**														
Behnoush et al. [50]	Y	Y	Y	Y	Y	Y	Y	Y	Y	Y	Y	Y	N	92%
Cruz & Morais [51]	Y	Y	NA	NA	N	N	Y	Y	NA	Y	NA	Y	N	67%
Vauclair et al. [52]	Y	N	Y	NA	NA	N	Y	Y	Y	Y	Y	Y	N	73%
**Wrist**														
Lendner et al. [53]	Y	N	Y	NA	NA	N	Y	N	Y	Y	Y	N	N	55%
Modest et al. [35]	N	N	Y	NA	NA	N	Y	Y	Y	Y	Y	Y	Y	73%
Pourahmadi et al. [54]	Y	Y	Y	Y	Y	N	Y	Y	Y	Y	Y	Y	Y	92%
Santos et al. [34]	Y	Y	NA	Y	Y	Y	NA	N	NA	Y	NA	Y	N	78%
**Hip**														
Charlton et al. [55]	Y	Y	N	NA	N	N	Y	Y	N	Y	Y	Y	N	58%
**Knee**														
Derhon et al. [56]	Y	Y	NA	Y	N	Y	NA	Y	NA	Y	NA	Y	Y	89%
Dos Santos et al. [57]	Y	Y	Y	Y	N	Y	Y	Y	Y	Y	Y	Y	Y	92%
Hambly et al. [58]	Y	Y	Y	NA	NA	N	Y	Y	Y	Y	Y	Y	Y	91%
Hancock et al. [59]	Y	Y	Y	Y	Y	Y	Y	Y	Y	Y	Y	Y	N	92%
Jones et al. [60]	Y	Y	Y	NA	NA	Y	Y	Y	Y	Y	Y	N	Y	91%
Mehta et al. [36]	Y	Y	Y	Y	Y	N	Y	Y	Y	Y	Y	Y	N	85%
Milanese et al. [1]	N	Y	Y	Y	Y	N	Y	Y	Y	Y	Y	Y	N	77%
Ockendon & Gilbert [61]	Y	N	Y	Y	Y	N	Y	Y	Y	Y	Y	Y	N	77%
Pereira et al. [37]	Y	Y	Y	Y	Y	Y	Y	Y		Y	Y	Y	N	85%
**Ankle**														
Morales et al. [62]	Y	Y	Y	Y	NA	N	Y	Y	Y	Y	Y	Y	Y	92%
Vohralik et al. [63]	Y	Y	Y	Y	Y	N	Y	Y	Y	Y	Y	Y	Y	92%
Williams et al. [64]	Y	Y	Y	NA	NA	Y	Y	Y	Y	Y	Y	Y	Y	91%
Number of studies that satisfied each criteria	35/37	31/37	27/30	24/25	18/26	16/37	30/31	33/37	27/30	37/37	28/30	33/37	16/37	

1 = If human subjects were used, did the authors give a detailed description of the sample of subjects used to perform the (index) test?; 2 = Did the authors clarify the qualification, or competence of the rater(s) who performed the (index) test?; 3 = Was the reference standard explained?; 4 = If interrater reliability was tested, were raters blinded to the findings of other raters?; 5 = If intrarater reliability was tested, were raters blinded to their own prior findings of the test under evaluation?; 6 = Was the order of examination varied?; 7 = If human subjects were used, was the time period between the reference standard and the index test short enough to be reasonably sure that the target condition did not change between the two tests?; 8 = Was the stability (or theoretical stability) of the variable being measured taken into account when determining the suitability of the time interval between repeated measures?; 9 = Was the reference standard independent of the index test?; 10 = Was the execution of the (index) test described in sufficient detail to permit replication of the test?; 11 = Was the execution of the reference standard described in sufficient detail to permit its replication?; 12 = Were withdrawals from the study explained?; 13 = Were the statistical methods appropriate for the purpose of the study?

N = No, R = Reliability, V = Validity; Y = Yes.

### Reliability and validity

The reliability and validity of the assessments are summarised in Table 3. For the sake of simplicity, the following three text sections will summarise the key results for intra-rater reliability, inter-rater reliability and validity, respectively.

**Table 3:** Reliability and validity of the selected studies.

Reference	Intra-rater reliability		Inter-rater reliability		Validity	
	Relative	Absolute	Relative	Absolute	Relative	Absolute
**Spine (trunk)**						
Bedekar et al. [38]	Trunk Flexion ICC = 0.920		Trunk Flexion ICC = 0.812		Trunk Flexion r = 0.95	
Furness et al. [39]	Thoracic rotation ICC = 0.94–0.98	Thoracic rotation SEM = 2.23° - 4.36° SRD = 8.74° - 17.09°	Thoracic rotation ICC = 0.72–0.89	Thoracic rotation SEM = 5.17° - 7.85° SRD = 14.33° - 21.76°	Thoracic rotation R^2^ = 0.697	Thoracic rotation LoA = 2.8° (-9.5° to 15.3°)
Grondin et al. [40]	Cervical Flexion-Rotation ICC = 0.95 Sagittal plane cervical ROM: ICC = 0.90	Cervical Flexion-Rotation SEM = 3.3° Flexion-Rotation Test: MDC^90^ = 7.6° Sagittal plane cervical ROM: SEM = 5.2° Sagittal plane cervical ROM: MDC^90^ = 12.2°				
Jung et al. [41]	Pelvic rotation ICC = 0.77–0.83	Pelvic rotation SEM = 0.64° - 0.73° MDC = 1.77° - 2.04°			Pelvic rotation ICC = 0.99	Pelvic rotation LoA = -1.16° (-2.2° to -0.12°)
Kolber & Hanney [42]	TCP Flexion ICC = 0.97 Lumbar Flexion ICC = 0.88 TCP Extension ICC = 0.80 TC Lateral Flexion ICC = 0.82–0.84		TCP Flexion ICC = 0.98 Lumbar Flexion ICC = 0.88 TCP Extension ICC = 0.81 TC Lateral Flexion ICC = 0.90–0.93	TCP Flexion MDC = 6° Lumbar Flexion MDC = 8° TCP Extension MDC = 9° TC Lateral Flexion MDC = 4°	TCP Flexion ICC = 0.97–0.98 Lumbar Flexion ICC = 0.86–0.87 TCP Extension ICC = 0.89–0.91 TC Lateral Flexion ICC = 0.91–0.96	TCP Flexion LOA = -15 to 15° Lumbar Flexion LOA = -7 to 18° TCP Extension LOA = -16 to 12° TC Lateral Flexion LOA -6 to 12°
Pourahmadi et al. [43]	Trunk Flexion ICC = 0.87–0.92 Trunk Extension ICC = 0.82–0.92.	Trunk Flexion: SEM = 2.1–3.0° trunk Extension: SEM = 2.3–2.74	Trunk Flexion ICC = 0.69–0.93 Trunk Extension ICC = 0.76–0.94	Trunk Flexion: SEM = 3.1° trunk Extension: SEM = 2.7°	Trunk Flexion: r = 0.85 Trunk Extension: r = 0.91	Trunk Flexion: LoA from -6.9° to 6.3° Trunk Extension: LoA from -5.4° to 4.9°
Pourahmadi et al. [30]	Cervical Flexion ICC = 0.76 Cervical Extension ICC = 0.76 Cervical Lateral Flexion ICC = 0.76–0.78 Cervical Rotation ICC = 0.70–0.78	Cervical Flexion SEM = 2.5° Cervical Extension SEM = 2.4° Cervical Lateral Flexion SEM = 1.0–1.4° Cervical Rotation SEM = 3.5–3.6° Cervical Flexion MDC = 6.9° Cervical Extension MDC = 6.7° Cervical Lateral Flexion MDC = 2.9–3.9° Cervical Rotation MDC = 9.8–9.9°	Cervical Flexion ICC = 0.65 Cervical Extension ICC = 0.67 Cervical Lateral Flexion ICC = 0. 71–0.76 Cervical Rotation ICC = 0. 76.0–79	Cervical Flexion SEM = 2.8° Cervical Extension SEM = 2.8° Cervical Lateral Flexion SEM = 1.5–2.1 Cervical Rotation SEM = 3.3–3.9° Cervical Flexion MDC = 7.7° Cervical Extension MDC = 7.6° Cervical Lateral Flexion MDC = 4.1–5.9° Cervical Rotation MDC = 9.1–9.7°	Cervical Flexion r = 0. 63 Cervical Extension r = 0.81 Cervical Lateral Flexion r = 0.72–0.79 Cervical Rotation r = 0.75–0.77	
Quek et al. [44]	Cervical Flexion ICC = 0.86 Cervical Extension ICC = 0.82 Cervical Lateral Flexion ICC = 0.85–0.90 Cervical Rotation ICC = 0.05–0.30	Cervical Flexion SEM = 3.1 Cervical Extension SEM = 5.0° Cervical Lateral Flexion SEM = 2.8–4.1° Cervical Rotation SEM = 15.8–16.4° Cervical Flexion MDC = 9.2° Cervical Extension MDC = 11.9° Cervical Lateral Flexion MDC = 8.3–12.2° Cervical Rotation MDC = 46.9–48.7°			Cervical Flexion ICC = 0.98 Cervical Extension ICC = 0.92 Cervical Lateral Flexion ICC = 0.95–0.96 Cervical Rotation ICC = 0.53	Cervical Flexion LOA = ± 2.3° Cervical Extension LOA = ± 9.6° Cervical Lateral Flexion LOA = ± 4.6–7.1° Cervical Rotation LOA = ± 9.6–18.6°
Stenneberg et al. [31]			Flexion-extension ICC = 0.90 Rotation ICC = 0.96 Lateral flexion ICC = 0.92	Flexion-extension LoA = (-11.97° to 15.19°) Rotation LoA = (10.06° to 13.82°) Lateral flexion LoA = (-10.95° to 9.93°)	Flexion-extension ICC = 0.95 Rotation ICC = 0.92 Lateral flexion ICC = 0.99	Flexion-extension LoA = 4.1° (-0.62° to 8.82°) Rotation LoA = 8.4° (2.7° to 14.14°) Lateral flexion LoA = 1.5° (-3.24° to 6.32°)
Tousignant-Laflamme et al. [45]	Examiner 1 with iPhone 4 Trunk Flexion ICC = 0.76 Trunk Extension ICC = 0.84 Lateral Flexion ICC = 0.77–0.78 Trunk Rotation ICC = 0.66–0.74 Examiner 2 with iPhone 3 GS Trunk Flexion ICC = 0.68 Trunk Extension ICC = 0.42 Lateral Flexion ICC = 0.68 Trunk Rotation ICC = 0.17–0.28		Trunk Flexion ICC = 0.48 Trunk Extension ICC = 0.49 Lateral Flexion ICC = 0.40–0.54 Trunk Rotation ICC = 0.07–0.07		Examiner 1 with iPhone 4 Trunk Flexion ICC = 0.76 Trunk Extension ICC = 0.58 Lateral Flexion ICC = 0.70–0.85 Trunk Rotation ICC = 0.43–0.55 Examiner 1 with iPhone 4 Trunk Flexion r = 0.69 Trunk Extension r = 0.56 Lateral Flexion r = 0.63–0.80 Trunk Rotation r = 0.38–0.58	
Ullucci et al. [46]	Cervical Right rotation IPhone: ICC = 0.98 Android: ICC = 0.91 Cervical Left rotation IPhone: ICC = 0.951 Android: ICC = 0.962		Peak ROM ICC = 0.87 Total ROM ICC = 0.82			
**Shoulder**						
Lim et al. [47]	Supine Shoulder Horizontal Adduction ICC = 0.72–0.89 Side Lying Shoulder Horizontal Adduction ICC = 0.95–0.97		Supine Shoulder Horizontal Adduction ICC = 0.79 Side Lying Shoulder Horizontal Adduction ICC = 0.94			
Mejia-Hernandez et al. [33]			Forward flexion ICC = 0.99 Total active abduction ICC = 0.99 Active glenohumeral Abduction ICC = 0.98 Passive glenohumeral Abduction ICC = 0.97 Active internal rotation ICC = 0.98 Passive internal rotation ICC = 0.98 Active external rotation ICC = 0.99 Passive external rotation ICC = 0.99			Forward flexion: LoA = −0.76° (−9.64° to 8.11°) Total active abduction LoA = 0.47° (−7.87° to 8.81°) Active glenohumeral Abduction LoA = -0.19° (−4.71° to 4.32°) Passive glenohumeral Abduction LoA = -0.38° (−4.02° to 3.25°) Active internal rotation LoA = 0.51° (−7.11° to 8.14°) Passive internal rotation LoA = 0.55° (−5.04° to 6.13°) Active external rotation LoA = -0.08° (−8.32° to 8.17°) Passive external rotation LoA = 0.4° (−7.58° to 8.37°)
Mitchell et al. [48]	Shoulder External Rotation ICC = 0.79		Shoulder External Rotation ICC = 0.94		Shoulder External Rotation ICC = 0.94	
Ramkumar et al. [49]						Flexion Mean difference = 4°± 2° Abduction Mean difference = 3°± 3° Internal rotation Mean difference = 2°± 4° External rotation Mean difference = 3°± 3°
Shin et al. [27]	Shoulder Flexion ICC = 0.96–0.99 Shoulder Abduction ICC = 0.96–0.99 Shoulder External Rotation ICC = 0.95–0.98 Shoulder Internal Rotation = 0.79–0.99	Second session, Observer A Shoulder Flexion SEM = 2.3–2.7° Shoulder Abduction SEM = 4.5–6.3° Shoulder External Rotation SEM = 2.8–3.3° Shoulder Internal Rotation SEM = 1.9–3.2°	Second session Shoulder Flexion ICC = 0.74–0.84 Shoulder Abduction ICC = 0.72–0.79 Shoulder External Rotation ICC = 0.76–0.90 Shoulder Internal Rotation ICC = 0.66–0.68	Second session Shoulder Flexion SEM = 9.6–10.1° Shoulder Abduction SEM = 13.2–13.8° Shoulder External Rotation SEM = 7.2–9.7° Shoulder Internal Rotation SEM = 10.5–10.6°	Shoulder Flexion ICC = 0.72–0.90 Shoulder Abduction ICC = 0.80–0.97 Shoulder External Rotation ICC = 0.89–0.97 Shoulder Internal Rotation ICC = 0.84–0.93	Shoulder Flexion LOA = 14–29° Shoulder Abduction LOA = 13–40° Shoulder External Rotation LOA = 10–18° Shoulder Internal Rotation LOA = 11–22°
Werner et al. [28]			Healthy: Shoulder Abduction ICC = 0.72 Shoulder Flexion ICC = 0.75 Shoulder External Rotation ICC = 0.86 Shoulder Internal Rotation ICC = 0.81 Symptomatic: Shoulder Abduction ICC = 0.91 Shoulder Flexion ICC = 0.97 Shoulder External Rotation ICC = 0.85–0.88 Shoulder Internal Rotation ICC = 0.86	Healthy: Shoulder Abduction SEM = 3.0° Shoulder Flexion SEM = 1.1° Shoulder External Rotation SEM = 3.7–4.0° Shoulder Internal Rotation SEM = 6.3° Symptomatic: Shoulder Abduction SEM = 0.3° Shoulder Flexion SEM = 3.3° Shoulder External Rotation SEM = 0.1–8.6° Shoulder Internal Rotation SEM = 5.1°	Healthy (as assessed by Orthopaedic Sports Medicine fellow) Shoulder Abduction ICC = 0.76 Shoulder Flexion ICC = 0.28 Shoulder External Rotation ICC = 0.66–0.78 Shoulder Internal Rotation ICC = 0.71 Symptomatic (as assessed by Orthopaedic Sports Medicine fellow) Shoulder Abduction ICC = 0.99 Shoulder Flexion ICC = 0.99 Shoulder External Rotation ICC = 0.96–0.97 Shoulder Internal Rotation ICC = 0.98	Healthy (as assessed by Orthopaedic Sports Medicine fellow) Shoulder Abduction MD ± LOA = 4.0 ± 9.5° Shoulder Flexion MD ± LOA = 6.2 ± 10.8° Shoulder External Rotation MD ± LOA = 6.9 ± 12.1° and 9.7 ± 14.9° Shoulder Internal Rotation MD ± LOA = 6.9 ± 10.9° Symptomatic (as assessed by Orthopaedic Sports Medicine fellow) Shoulder Abduction MD ± LOA = 2.6 ± 4.1° Shoulder Flexion MD ± LOA = 2.4 ± 4.3° Shoulder External Rotation MD ± LOA = 2.0 ± 3.4° and 2.5 ± 5.8° Shoulder Internal Rotation MD ± LOA = 1.7 ± 4.0°
**Elbow**						
Behnoush et al. [50]			Elbow flexion: ICC = 0.95 Pronation: ICC = 0.98 Supination: ICC = 0.98		Elbow flexion: ICC = 0.84 Pronation: ICC = 0.90 Supination: ICC = 0.96	Elbow Flexion MD ± LoA = -0.4° (-3.9° to 3.0°) Pronation MD ± LoA = 0.4° (-5.8° to 5.0°) Supination MD ± LoA = -0.4° (-6.0° to 5.2°)
Cruz & Morais [51]		Elbow flexion at onset of pain dominant side: SEM = 6.6° Elbow flexion at onset of pain non-dominant side: SEM = 6.8° Elbow flexion at onset of pain dominant side: MDC^95^ = 18.4° Elbow flexion at onset of pain non-dominant side: MDC^95^ = 18.8° Elbow flexion at max tolerable pain dominant side: SEM = 4.8° Elbow flexion at max tolerable pain non-dominant side: SEM = 4.2° Elbow flexion at max tolerable pain dominant side: MDC^95^ = 13.2° Elbow flexion at max tolerable pain non-dominant side: MDC^95^ = 11.7°				
Vauclair et al. [52]						Flexion SEM = 1° Extension SEM = 0.8° Flexion SEM = 1.9° Flexion SEM = 1.2°
**Wrist**						
Lendner et al. [53]						Wrist Range of Motion MD (LoA) = 0.5°. (-16.7° to 17.7°)
Modest et al. [35]					Healthy: Wrist flexion ICC = 0.97 Wrist extension ICC = 0.96 Wrist supination ICC = 0.95 Wrist pronation ICC = 0.96 Injured: Wrist flexion: ICC = 0.99 Wrist extension ICC = 0.99 Wrist supination ICC = 0.99 Wrist pronation ICC = 0.99	LoA = average absolute deviation < 2°
Pourahmadi et al. [54]	Within day Wrist Flexion ICC = 0.89 Wrist Extension ICC = 0.90 Wrist Radial Deviation ICC = 0.87 Wrist Ulnar Deviation ICC = 0.91	Within day Wrist Flexion SEM = 1.6° Wrist Extension SEM = 1.0° Wrist Radial Deviation SEM = 0.9° Wrist Ulnar Deviation SEM = 1.1° Wrist Flexion MDC = 4.3° Wrist Extension MDC = 2.9° Wrist Radial Deviation MDC = 2.6° Wrist Ulnar Deviation MDC = 3.1°	Wrist Flexion ICC = 0.79 Wrist Extension ICC = 0.81 Wrist Radial Deviation ICC = 0.80 Wrist Ulnar Deviation ICC = 0.82	Wrist Flexion SEM = 2.2° Wrist Extension SEM = 1.7° Wrist Radial Deviation SEM = 1.1° Wrist Ulnar Deviation SEM = 1.6° Wrist Flexion MDC = 6.2° Wrist Extension MDC = 4.6° Wrist Radial Deviation MDC = 2.9° Wrist Ulnar Deviation MDC = 4.5°	Wrist Flexion r^2^ = 0.70 Wrist Extension r^2^ = 0.63 Wrist Radial Deviation r^2^ = 0.73 Wrist Ulnar Deviation r^2^ = 0.84	Wrist Flexion MD (LOA) -0.9 (-6.1 to 4.2°) Wrist Extension MD (LOA) -0.6 (-5.6 to 4.5°) Wrist Radial Deviation MD (LOA) -0.5 (-3.3 to 2.3°) Wrist Ulnar Deviation MD (LOA) -1.0 (-3.9 to 1.9°)
Santos et al. [34]	Injured i-Phone 5 with selfie-stick Wrist Pronation and Supination: ICC = 0.94–0.96 i-Phone 5 handheld Wrist Pronation and Supination: ICC = 0.94–0.95 Non-injured i-Phone 5 with selfie-stick Wrist Pronation and Supination: ICC = 0.89–0.93 i-Phone 5 handheld Wrist Pronation and Supination: ICC = 0.77–0.92		Injured i-Phone 5 with selfie-stick Wrist Pronation and Supination: ICC = 0.94 i-Phone 5 handheld Wrist Pronation and Supination: ICC = 0.92 Non-injured i-Phone 5 with selfie-stick Wrist Pronation and Supination: ICC = 0.89 i-Phone 5 handheld Wrist Pronation and Supination: ICC = 0.72	Injured i-Phone 5 with selfie-stick Wrist Pronation and Supination SEM = 3.7° i-Phone 5 handheld Wrist Pronation and Supination SEM = 3.3° Non-Injured i-Phone 5 with selfie-stick Wrist Pronation and Supination SEM = 4.1° i-Phone 5 handheld Wrist Pronation and Supination SEM = 3.8°		i-Phone 5 with selfie-stick vs pencil goniometer Wrist Pronation and Supination LOA = -15° to 15° i-Phone 5 handheld vs bubble goniometer Wrist Pronation and Supination: LOA = -10° to 10°
**Hip**						
Charlton et al. [55]	Hip Flexion ICC = 0.86 Hip Abduction ICC = 0.68 Hip Adduction ICC = 0.68 Hip Supine IR ICC = 0.94 Hip Supine ER ICC = 0.87 Hip Sitting IR ICC = 0.84 Hip Sitting ER ICC = 0.63	Hip Flexion SEM = 2.3° Hip Abduction SEM = 4.6° Hip Adduction SEM = 2.5° Hip Supine IR SEM = 3.2° Hip Supine ER SEM = 2.6° Hip Sitting IR SEM = 3.4° Hip Sitting ER SEM = 2.8°			Hip Flexion ICC = 0.92 Hip Abduction ICC = 0.98 Hip Adduction ICC = 0.91 Hip Supine IR ICC = 0.88 Hip Supine ER ICC = 0.71 Hip Sitting IR ICC = 0.92 Hip Sitting ER ICC = 0.90	
**Knee**						
Derhon et al. [56]	Knee flexion ICC = 0.83–0.86	Knee flexion LoA = 1.1° (-10.7° to 12.8°) to 2.5° (-7.9° to 12.9°)	Knee flexion ICC = 0.89–0.95	Knee flexion LoA = 0° (-4.9° to 5°) to -2° (-8.3° to 4.3°)		
Dos Santos et al. [57]					Knee flexion ICC = 0. 0.88–0.96	Knee flexion CoV: 22.3% - 26.4%
Hambly et al. [58]					Knee Flexion r = 0.93 Knee Flexion ICC = 0.89	Knee Flexion MD ± LoA = 1.3° (-2.1° to 4.9°)
Hancock et al. [59]	Knee flexion ICC = 0.99	Knee flexion SEM = 11.72°	Knee flexion ICC = 0.99			
Jones et al. [60]	Knee Flexion ICC = 0.96–0.98 (single measures) Knee Flexion ICC = 0.98–0.99 (average of measures)	Knee Flexion Measurement 1 SDMD = 2.6° and SEMD = 0.4° Knee Flexion Measurement 2 SDMD = 3.3 and SEMD = 0.6° Knee Flexion Measurement 3 SDMD = 2.3 and SEMD = 0.4°			Knee Flexion r = 0.96–0.98	Knee Flexion Measurement 1 MD ± LoA = 0.5° (-4.6° to 5.6°) Knee Flexion Measurement 2 MD ± LoA = 0.5° (-6.0° to 7.1°) Knee Flexion Measurement 3 MD ± LoA = 0.5° (-4.0° to 5.1°)
Mehta et al. [36]	Knee Flexion ICC = 0.97 Knee Extension ICC = 0.94	Knee flexion SEM = 2.72° Knee extension SEM = 1.18° Knee flexion MDC^90^ = 6.3° Knee extension MDC^90^ = 2.7°	Knee flexion ICC = 0.99 Knee extension ICC = 0.97		Knee flexion r = 0.92 Knee extension r = 0.68	Flexion MD ± LoA = 4.97 (-7.3° to 17.3°) Extension MD ± LoA = 0.98 (-6.6° to 8.5°)
Milanese et al. [1]	Knee flexion 3 Clinicians: CCC = 0.99 Knee flexion 3 Students CCC = 0.99	Knee flexion 3 Clinicians: SEM = 1.4° Knee flexion 3 Students SEM = 1.5°	Knee flexion CCC = 0.99		Knee flexion 3 Clinicians: CCC = 0.98 Knee flexion 3 Students CCC = 0.98	
Ockendon & Gilbert [61]	Knee flexion r = 0.99		Knee flexion r = 0.98		Knee flexion r = 0.95	Knee flexion MD ± LoA = -0.4° ± 3.9°
Pereira et al. [37]	*Postoperative* Knee flexion active motion ICC = 0.99 Knee flexion passive motion: ICC = 0.92 *Healthy* Knee flexion active motion ICC = 0.86 Knee flexion passive motion: ICC = 0.90		*Postoperative* Knee flexion active motion ICC = 0.43 Knee flexion passive motion: ICC = 0.27 *Healthy* Knee flexion active motion ICC = 0.12 Knee flexion passive motion: ICC = 0.13		*Postoperative* Knee Active extension CCC = 0.80 Knee Active flexion CCC = 0.97 Knee Passive extension CCC = 0.72 Knee Passive flexion CCC = 0.99 *Healthy* Knee Active extension CCC = 0.88 Knee Active flexion CCC = 0.60 Knee Passive extension CCC = 0.90 Knee Passive flexion CCC = 0.50	
**Ankle**						
Morales et al. [62]	Ankle dorsiflexion ICC = 0.97–0.98	Ankle dorsiflexion SEM = 0.29 cm—0.43 cm MDC = 0.79 cm—1.19 cm				
Vohralik et al. [63]	Ankle Dorsiflexion ICC = 0.97	Ankle Dorsiflexion SEM = 1.4°	Ankle Dorsiflexion ICC = 0.76	Ankle Dorsiflexion SEM = 3.4°	Ankle Dorsiflexion r^2^ = 0.99	Ankle dorsiflexion MD ± LoA = ~0.5^o^ (-0.8 to 1.8^o^)
Williams et al. [64]	Ankle dorsiflexion with straight knee ICC = 0.81 Ankle dorsiflexion with bent knee ICC = 0.85		Ankle dorsiflexion with straight knee ICC = 0.80 Ankle dorsiflexion with bent knee ICC = 0.96		On identical hard surfaces in multiple planes ICC = 0.99	On identical hard surfaces in multiple planes LoA = (-4.1 to 5.0^0^)

CCC = Concordance correlation coefficient, Coefficient of Variation = CoV, ER = external rotation, ICC = intraclass correlation, IR = internal rotation, LoA = limits of agreement, MD = mean difference, MDC = minimal detectable change, r = Pearson’s product moment correlation, SDMD = Standard deviation of the mean difference, SEM = standard error of the measurement, SEMD = standard error of the mean difference, SRD = standard real difference, TCP = thoracolumbar-pelvic.

#### Intra-rater reliability

Twenty-six studies assessed aspects of intra-rater reliability, with 10 studies reporting relative metrics only, one study reporting absolute metrics only, and the remaining 15 studies reporting both relative and absolute metrics. Twenty-five of 26 studies reported excellent intra-rater relative reliability as defined by an ICC > 0.75 for more than 50% of the joint movements they examined, the only exception being Tousignant-Laflamme et al. [45]. However, this classification of poor relative intra-rater reliability for Tousignant-Laflamme et al. [45] was primarily due to the results of one examiner using an iPhone 3, compared to the other examiner who used an iPhone 4. If we were to consider all the studies that assessed relative intra-rater reliability with an iPhone 4, all six studies demonstrated that smartphone apps had adequate relative intra-rater reliability [1, 34, 37, 42, 48, 64]. Thirteen of 17 studies reported good absolute intra-rater reliability as defined by a SEM or MDC < 5^o^ or LOA < ± 9.8^o^ for more than 50% of the joint movements they examined, with only four studies not satisfying this threshold [40, 44, 51, 59]. It should however be noted that the study by Quek et al. [44] satisfied the criteria for more than 50% of the movements when quantified by the SEM (the three of the four movements) but failing this when reliability was assessed by the MDC for all four movements.

#### Inter-rater reliability

Twenty-five studies assessed aspects of inter-rater reliability, in which 13 studies reported relative metrics only, and 12 studies reported both relative and absolute metrics. Twenty-three of 25 studies demonstrated excellent inter-rater reliability as defined by an ICC > 0.75 for more than 50% of the joint movements they examined, with only two studies not satisfying this threshold for relative inter-rater reliability [37, 45]. Six out of 11 studies reported good absolute inter-rater reliability as defined by a SEM or MDC < 5^o^ or LOA < ± 9.8^o^ for more than 50% of the joint movements they examined, with five studies not satisfying this criteria [27, 30, 31, 39, 42]. While Pourahmadi et al. [30] was deemed to not meet this threshold of absolute inter-rater reliability, this was based on all four MDC values > 5^o^, although the SEM values for the same movements were all < 5^o^.

#### Validity

Thirty studies measured some aspects of validity, of which seven studies reported relative metrics only, five studies reported absolute metrics only, and 18 studies reported both relative and absolute metrics. Twenty of 25 studies observed excellent/substantial relative validity as defined by ICC > 0.75, r > 0.9 or CCC > 0.95 for more than 50% of the joint movements examined, with five studies not meeting this threshold for this criteria [28, 30, 36, 45, 54]. Seventeen of 23 studies observed excellent/substantial absolute validity as defined by SEM or MDC < 5^o^ or LOA < ± 9.8^o^ for more than 50% of the joint movements they examined, with six studies not meeting this threshold of absolute validity [27, 34, 36, 42, 53, 57].

## Discussion

This study systematically reviewed the literature for studies which examined the reliability and/or validity of smartphones and apps to quantify joint ROM. Thirty-seven studies were found to be eligible, with the studies assessing joint ROM across most of the body’s major joints. Specifically, the most common joints assessed were the spine/trunk (n = 11), knee (n = 9) and shoulder (n = 6), with a smaller number of studies examining the wrist (n = 4), elbow (n = 3), ankle (n = 3) and hip (n = 1) joints. The primary result of the systematic review was that the apps generally demonstrated adequate intra-rater and inter-rater reliability as well as validity when compared to criterion devices such as goniometers, inclinometers and 3D motion capture. However, there was a trend for the reliability outcomes that these results were somewhat stronger for relative (e.g. ICC, r) than absolute measures (e.g. SEM, MDC).

The tendency for the relative measures to be stronger than absolute measures is something that needs to be clearly understood by the clinician. Historically, many reliability and/or validity studies have only reported relative statistics such as the ICC and Pearson’s product moment correlation [3, 7, 8]. Relative statistical measures are typically used to describe the resemblance of two or more units within a group (e.g. the similarity of measurements undertaken by two clinicians) as a function of the resemblance between different groups. ICC is thus operationalized as a ratio between two variance measures [66]. To illustrate, the inter-rater reliability ICC measure of Pourahmadi et al. [30] is derived by the ratio of variance between (1) the variance between two measurements from the same participant, repetition, and session, against (2) the variance between two measurements from the same participant, repetition, session, from different raters [66]. While these relative statistics provide important information regarding the correlation or rank order of two or more measurements, they provide no detail regarding the magnitude of change/difference in the measurement across these time points [20, 21]. In contrast, absolute statistical measures of reliability/validity simply report the resemblance of two or more units within a group–in other words, it simply represents the individual variance components [66]. Clinically, an ICC is useful for a manager wanting to train a team of clinicians in the use of a mobile app, where the aim is to achieve a value as close to one. However, the individual variance components of between repetition and between sessions are more useful for the day-to-day practice of individual clinicians. Knowing the inherent variation in outcomes between each measurement repetition and between clinical visits, allows a clinician to judge the clinical importance of any kinematic change using a mobile app.

With respect to the validity of smartphones and apps to quantify ROM, it was apparent that the majority of studies included in this review assessed the validity of the smartphone app against a universal goniometer as the criterion test. However, it could be argued that the most appropriate criterion measure to determine joint ROM would be radiographic images such as x-ray or 3D motion capture. Only five studies utilised 3D motion capture as the criterion method [31, 41, 44, 55, 63]. All five of these studies demonstrated that the apps had adequate levels of relative validity with respect to 3D motion capture [31, 41, 44, 55, 63], with a similar result observed for all of the three studies assessing absolute metrics also reporting adequate validity [31, 41, 44]. It should also be noted that Charlton et al. [55] compared the relative validity of their smartphone app and inclinometer to the criterion method of 3D motion capture for assessing hip joint ROM. Based on the ICC threshold of 0.75 for sufficient validity, both devices were valid, with the smartphone exceeding this threshold for five of the six joint ROM and the inclinometer for all six. The use of 3D motion capture as a criterion measure may be more important when assessing dynamic rather than static joint ROM due to the inherent difficulties in maintaining correct position of the universal goniometer on the joint centre and its alignment with the proximal and distal joints during movement, especially at high movement velocities [11]. All the five 3D motion capture validity studies included in the present review assessed static ROM [31, 41, 44, 55, 63]–i.e. when range is recorded when a joint was positioned statically at its limit of motion. The lack of assessment of apps on dynamic ROM may not be surprising given that assessors need a joint to be held transiently in a static position to record the range from the app. For apps to measure dynamic ROM, it needs to sample a joint’s motion throughout the movement task, and this data needs to be post-processed to extract parameters of ROM–similar to how a 3D motion capture system quantifies ROM. Future studies are warranted to quantify the validity and reliability of smartphone apps in the assessment of dynamic ROM.

Another issue of major importance to clinicians is whether the smartphones and apps display adequate reliability and validity across all joints, joint actions and populations. It was heartening to see that most of these variables did not seem to influence the reliability and validity of the apps in measuring joint ROM. There was clear variation in the reliability and validity in different spinal joint movements, as well as a tendency for differences in reliability and validity between healthy and clinical populations and to a lesser extent smartphone models that the clinician should be aware.

When examining the 11 studies examining spinal ROM, it appeared that the assessment of flexion, extension and lateral flexion typically exhibited adequate relative reliability and/or validity [30, 31, 38–46], although not all of these studies assessed absolute reliability and validity. Compared to the assessment of spinal flexion, extension and lateral flexion, the assessment of spinal axial rotation did not exhibit adequate reliability and validity in four [30, 40, 44, 45] of the nine studies. On this basis, it would appear that while the apps used within the studies reviewed in this manuscript typically had adequate reliability and validity for measuring spinal flexion, extension and lateral flexion, they are somewhat more questionable for measuring spinal rotation. Nevertheless, a recent study by Furness et al. [5] demonstrated comparable (or slightly better) reliability of an iPhone 6 and the Compass app to the universal goniometer for assessing thoracic rotation in healthy individuals. Unfortunately, while this study also demonstrated strong correlations between the Compass app and the universal goniometer, absolute validity was again inadequate as the limits of agreement between the two devices was ~25° [5]. Such findings suggest that the ability to perform a valid assessment of spinal rotation using devices that are feasible in clinical practice, be it goniometers or smartphone-based apps, may still remain somewhat questionable. Further research and/or additional clinical training into the use of these devices in this context is therefore warranted.

The comparatively poorer reliability and validity of smartphone apps measuring ROM in axial rotation compared to flexion-extension and lateral flexion could be attributed to several factors. First is the difference in smartphone sensor performance in different Cardinal planes [67]. Using performance testing of commercial Inertial Measurement Units (IMUs) as an example, the static error of the Xsens MT9 IMU was three times greater in the yaw (axial rotation) direction, than in the other two Cardinal Planes [68]. Second, is the reliance of different components of the smartphone sensor (e.g. magnetometer vs gyroscope) when measuring ROM in different Cardinal planes. Magnetometers are required when testing axial rotation in an anti-gravity position (e.g. sitting) [44, 45]. Compared to gravity-dependent gyroscopes, magnetometers are more sensitive to signal distortion arising from the environmental magnetic fields, potentially reducing their validity and reliability. In contrast, Pourahmadi et al. [30] tested cervical rotation in supine using the gravity-dependent gyroscope component of the smartphone sensor. This could explain the better validity and reliability of Pourahmadi et al. [30] compared to two other studies who reported poor reliability and validity [44, 45]. Third, is the issue of axis mis-alignment which occurs when the sensor’s coordinate axes are not aligned with anatomically meaningful axes [69]. There may be greater potential for axis mis-alignment, during axial rotation than in other movement directions [70–72]. Given that spinal axial rotation commonly couples with secondary movement in other directions, maintaining a pure axial rotation may be difficult.

While most of the studies reviewed in this manuscript involved healthy participants, some recruited patients with joint pain. These studies included groups of individuals with neck pain [30, 31], shoulder pathology [27, 32, 33], various upper limb injuries [34, 35] or knee pain [36, 37]. The intra-rater and inter-rater reliability of the apps in these clinical populations was typically adequate in these nine studies, with the exception of Pereira et al. [37]. The validity of the apps in these populations was sufficiently high in six of the nine studies. For the three studies with insufficient validity [34, 36, 37], a variety of statistical approaches were used, with the results being CCC = 0.50–0.72, r = 0.68 and LoA ranging from -10 to +17.3^o^ for the measured joint actions. Such results may suggest that using smartphones and apps can be quite reliable in a range of population groups, including some clinical populations presenting with musculoskeletal pathology.

The clinician should also be aware of the potential for how the make and model of the smartphone and the actual app can influence the reliability of assessment and how these two factors; as well as how the criterion test selected may influence the validity. While there was some variability between studies in the smartphone used (29 studies using iPhones, most commonly iPhone 4 or 5), there was little evidence of any effect of smartphone with the exception of one study [45]. Specifically, Tousignant-Laflamme et al. [45] reported adequate relative intra-rater reliability for an examiner with an iPhone 4, but not an examiner with an iPhone 3; with this ultimately resulting in poor inter-rater reliability. Further, the two examiners were unable to demonstrate adequate validity when compared to the CROM device, which is considered a criterion measure for measuring cervical ROM. Such results suggest that clinicians should use more recently developed smartphones, which are more likely to have improved sensor capacity than older smartphone models such as the iPhone 3.

With respect to the number of apps included in this review, there was a wide variety examined in these 37 studies. This was clearly demonstrated as only two apps were used in more than two studies, these being the Clinometer (n = 5) and Knee Goniometer (n = 3). The wide diversity of apps utilised in these studies and the general support for all of these apps’ reliability and validity demonstrated in this review, suggest that the clinician has multiple options when selecting the most appropriate app for measuring a particular joint ROM. However, it would still be recommended that clinicians utilise apps that have been demonstrated to be reliable and valid for measuring the particular joint action they wish to measure. We would also recommend that researchers need to continue to examine the reliability and validity of more recently developed apps and smartphones to determine if they offer advantages over those previously developed and assessed in the scientific literature.

This systematic review has several strengths and limitations that need to be acknowledged. A primary strength of this review in comparison to the literature [14, 15] is that it provides more detailed reporting of key aspects of the methodology and the actual relative and absolute intra-rater, inter-rater and validity outcomes for each joint action assessed in each study within our summary tables. The current study also appears to be the first systematic review on this topic to use a validated tool to assess the included studies’ methodological quality. By performing this assessment of study quality, it was determined that only two of the 37 studies were considered to be of low quality, based on a CAT score of less than 60% [53, 55]. Further, only two of the 13 CAT criteria were achieved in less than 50% of the studies (Criteria Six: Order of Examination and Criteria 13: Statistical Methods). The low score for Criteria Six: Order of Examination reflected the lack of randomisation and the potential for a learning or fatigue effect in many of the studies. The low score for Criteria 13 (Statistical Methods) tended to reflect the fact that most studies only reported relative reliability and/or validity statistics (e.g. r or ICC) without also reporting comparable absolute reliability and/or validity statistics (e.g. SEM, MDC or MD±LOA). As each of these three CAT criteria are highly important characteristics of strong psychometric study design, improvement in these areas would further strengthen the level of evidence described in this review.

The primary limitation of our review process reflected the manner in which sufficient validity and reliability was described. Specifically, we utilised a process in which a particular app was described as suitably reliable and/or valid when recommended statistical thresholds were achieved in more than 50% of the movements examined in each study. While this approach is useful as a generalised approach to describe the reliability and/or validity of an app, it is perhaps a little bit too simplistic due to the relatively high between-study variation in populations, joints, joint actions, smartphone and app (including software updates). This potential negative influence of software updates on the reliability and validity of apps has also been recently highlighted as a major issue in the use of global positioning systems (GPS) in sport [73]. Due to the limitation of our somewhat arbitrary greater than 50% reliability and validity threshold, we would suggest that clinicians should still examine the actual data summarised in this systematic review, as it is quite possible that different joint motions may demonstrate differences in their reliability and validity, even when assessed in the same population with the same smartphone and app. The final limitation of this systematic review is that we cannot be 100% certain that all eligible articles were identified and included in this systematic review.

### Conclusion

The results of this systematic review provide relatively strong evidence regarding the intra-rater, inter-rater and validity of smartphones and apps to assess joint ROM; with these results tending to be observed across multiple joints, joint actions, populations, smartphones and apps. Such results suggest that clinicians may be able to use a relatively wide variety of smartphones and apps to quantify joint ROM. However, when absolute validity was assessed, they were often reasonably large differences in the angle determined by an app compared to a criterion measure such as 3D motion capture, goniometry or inclinometers. On this basis, it is imperative that the clinician does not switch between different assessment devices (such as a goniometer and a smartphone based apps) when assessing an individual across multiple time points. Clinical researchers should also aim to develop more reliable and valid protocols for using smartphones and apps, while continuing to collaborate with smartphone and app developers to further improve their reliability and validity for assessing joint ROM.

## Supporting information

S1 Checklist PRISMA checklist. (DOC)

S1 Appendix PRISMA flowchart. (DOCX)
