# Supplementary material for: Reliability and validity of clinically accessible smartphone applications to measure joint range of motion: A systematic review
Source: PLoS One. 2019 May 8;14(5):e0215806. doi: 10.1371/journal.pone.0215806 (PMC6505893; doi:10.1371/journal.pone.0215806)
Supplement: S1 Appendix — (DOCX) [file pone.0215806.s002.docx]

The search strategy that was conducted involved three subjects headings: range of motion (“range of motion”, “joint range”, “joint angle”, “angular measurement”, “joint movement”, “goniometer”); smartphone (“smartphone”, “smartphones”, “smartphone application”, “applications”, “app”, “apps”, “mobile phone”, “mobile device”, “iphone”, “handheld device”, “phone”, “compass”, “inclinometer”); and reliability and validity (“validity”, “valid”, “validation”, “reliability”, “reliable”). The Boolean operator “OR” was used within subject headings and the Boolean operator “AND” between subject headings. Studies were identified if they had a word from each subject heading in their title or abstract.
